# Polymorphisms in thymidylate synthase gene and susceptibility to breast cancer in a Chinese population: a case-control analysis

**DOI:** 10.1186/1471-2407-6-138

**Published:** 2006-05-25

**Authors:** Xiangjun Zhai, Jun Gao, Zhibin Hu, Jinhai Tang, Jianwei Qin, Shui Wang, Xuechen Wang, Guangfu Jin, Jiyong Liu, Wenshen Chen, Feng Chen, Xinru Wang, Qingyi Wei, Hongbing Shen

**Affiliations:** 1 Laboratory of Reproductive Medicine, Nanjing Medical University, Nanjing 210029, China; 2 Department of Epidemiology and Biostatistics, School of Public Health, Nanjing Medical University, Nanjing 210029, China; 3 Jiangsu Centers for Diseases Prevention and Control, Nanjing 210009, China; 4 Department of General Surgery, Jiangsu Cancer Hospital, Nanjing 210009, China; 5 Department of General Surgery, The First Affiliated Hospital of Nanjing Medical University, Nanjing 210029, China; 6 Department of General Surgery, Nanjing Gulou Hospital, Nanjing210009, China; 7 Department of Epidemiology, The University of Texas M. D. Anderson Cancer Center, Houston, TX 77030, USA

## Abstract

**Background:**

Accumulative evidence suggests that low folate intake is associated with increased risk of breast cancer. Polymorphisms in genes involved in folate metabolism may influence DNA methylation, nucleotide synthesis, and thus individual susceptibility to cancer. Thymidylate synthase (TYMS) is a key enzyme that participates in folate metabolism and catalyzes the conversion of dUMP to dTMP in the process of DNA synthesis. Two potentially functional polymorphisms [a 28-bp tandem repeat in the *TYMS *5'-untranslated enhanced region (*TSER*) and a 6-bp deletion/insertion in the *TYMS *3'-untranslated region (*TS 3'-UTR*)] were suggested to be correlated with alteration of thymidylate synthase expression and associated with cancer risk.

**Methods:**

To test the hypothesis that polymorphisms of the *TYMS *gene are associated with risk of breast cancer, we genotyped these two polymorphisms in a case-control study of 432 incident cases with invasive breast cancer and 473 cancer-free controls in a Chinese population.

**Results:**

We found that the distribution of *TS3'-UTR *(1494del6) genotype frequencies were significantly different between the cases and controls (*P *= 0.026). Compared with the *TS3'-UTR del6/del6 *wild-type genotype, a significantly reduced risk was associated with the *ins6/ins6 *homozygous variant genotype (adjusted OR = 0.58, 95% CI = 0.35–0.97) but not the *del6/ins6 *genotype (OR = 1.09, 95% CI = 0.82–1.46). Furthermore, breast cancer risks associated with the *TS3'-UTR del6/del6 *genotype were more evident in older women, postmenopausal subjects, individuals with a younger age at first-live birth and individuals with an older age at menarche. However, there was no evidence for an association between the *TSER *polymorphism and breast cancer risks.

**Conclusion:**

These findings suggest that the *TS3'-UTR del6 *polymorphism may play a role in the etiology of breast cancer. Further larger population-based studies as well as functional evaluation of the variants are warranted to confirm our findings.

## Background

Breast cancer is the leading cause of cancer-related deaths for women in China, and the incidence rate is still increasing [1]. A wide diversity of genetic damage induced by endogenous metabolites and exogenous hazards may contribute to the etiology of breast cancer. Epidemiological studies suggested that folate-deficient diet may increase the risk for breast cancer [2-5]. Folate, often in the form of folic acid, is essential for DNA methylation, DNA synthesis, and DNA repair [6,7]. Chronic folate/methyl deficiency *in vivo *and *in vitro *has been associated with DNA strand breaks [8-10] and abnormal DNA methylation [11,12] that may lead to carcinogenesis.

Many enzymes are involved in folate metabolism, among which, thymidylate synthase (TYMS) catalyzes the conversion of deoxyuridine monophosphate (dUMP) to deoxythymidine monophosphate (dTMP), using the 5, 10-methylenetetrahydrofolate as a methyl donor [13]. This conversion is essential for the provision of thymidine, a nucleotide needed for DNA synthesis and DNA repair [14]. In thymidylate synthase-negative mutants of mouse FM3A cells, thymidine starvation results in thymineless death via the induction of DNA double-strand breaks that lead to chromosome fragmentation as well as rearrangements in the cells synthesizing DNA [15]. Therefore, genetic alterations in TYMS enzyme efficiency and/or expression level may contribute to individual susceptibility to breast cancer.

The *TYMS *gene is located in chromosome 18p11.32. A tandem repeat polymorphism has been identified in the *5'-UTR *enhancer region of the *TYMS *promoter (*TSER*), the immediate upstream of the ATG codon initiation start site, which contains triple (*TSER 3R*) or double (*TSER 2R*) repeats of a 28-bp sequence as well as several rare alleles containing 4, 5 or 9 repeats [16]. *In vitro *and *in vivo *studies showed that TYMS expression was *TSER *genotype-dependent and that the *3R *allele was associated with a higher TYMS expression level [16,17]. Recently, a novel polymorphism, a 6-bp deletion/insertion in the 3'-untranslated region of the *TYMS *gene (*TS3'-UTR del6*), has also been identified, which may have an effect on the *TYMS *mRNA stability and translation [18], perhaps affecting TYMS protein expression level as well. Therefore, the above two *TYMS *variants are thought to be functionally relevant and are hypothesized to be associated with risk of breast cancer. To test this hypothesis, we performed genotyping analyses for *TS 5'-UTR TSER *and *TS 3'-UTR del6 *polymorphisms in a case-control study of 432 incident cases with invasive breast cancer and 473 cancer-free controls in a Chinese population.

## Methods

### Study population

This hospital-based case-control study consisted of 432 patients who received surgical treatment for solitary mammary lumps, which were histopathologically diagnosed as invasive breast cancer, and 473 cancer-free controls. All subjects were genetically-unrelated ethnic Han Chinese and were from Nanjing city and surrounding regions in Jiangsu Province. The breast cancer patients were consecutively recruited between January 2004 and July 2005 at the Cancer Hospital of Jiangsu Province, the First Affiliated Hospital of Nanjing Medical University and the Nanjing Gulou Hospital, Nanjing, China. Those patients who had any previous cancers, *in situ *breast cancer (DCIS), metastasized cancer originated from other organs, and previous radiotherapy or chemotherapy were excluded. Cancer-free controls were randomly selected from a pool of 10,500 individuals who participated in a community-based screening program for non-infectious diseases conducted in Jiangsu Province during the same time period as the cases were recruited. These control subjects had no self-reported cancer history and were frequency-matched to the cases on age (± 5 years) and residential areas. Each subject was scheduled for an interview after an informed consent was obtained, and a structured questionnaire was administered by interviewers to collect information on demographic data, menstrual and reproductive history, family history of cancer (any kind of cancers in first-degree relatives) and environmental exposure history. After the interview, an approximately 5-ml venous blood sample was collected from each subject. The study was approved by the institutional review board of Nanjing Medical University.

### Genotype analyses

By centrifugation of 5-ml whole blood, genomic DNA was extracted from the leukocyte pellet obtained from the buffy coat of each blood sample. We used the previously-described genotyping assays for the two loci of *TYMS *[19]. Briefly, we used pairs of primers of 5'-GTGGCTCCTGCGTTTCCCCC-3' (forward) and 5'-GGCTCCGAGCCGGCCACAGGCATGGCGCGG-3' (reverse) to genotype the *TSER *locus and 5'-CAAATCTGAGGGAGCTGAGT-3' (forward) and 5'-CAGATAAGTGGCAGTACAGA-3' (reverse) to genotype the *TS 3'-UTR del6 *locus. For the *TSER *polymorphism, 243 bp (i.e. 3R), 215 bp (i.e. 2R), 271 bp (i.e. 4R) and 299 bp (i.e. 5R) fragments were identified and separated on 3% agarose gels. For the *TS 3'-UTR del6 *polymorphism, 152 bp (i.e. del6) or 158 bp fragments (i.e. ins6) were amplified and then digested by *Dra *I (New England BioLabs, Inc., Beverly, MA). The variant (ins6) allele produces two fragments of 88 and 70 bp, while the wild-type (del6) allele produces a single 152-bp fragment. Genotyping was performed without knowing the subjects' case or control status, and approximately equal number of cases and controls were assayed in each 96-well PCR plate with a positive control of a DNA sample with a known heterozygous genotype. If a consensus on the tested genotype was not reached, two research assistants independently performed the repeated assays to achieve 100% concordance. More than 10% of the samples were repeated using the same assay and the results were 100% concordant.

### Statistical analyses

Differences in demographic characteristics, selected variables, and frequencies of the genotypes, alleles, and haplotypes of *TSER *and *TS 3'-UTR del6 *between the cases and controls were evaluated using the χ^2 ^test (for categorical variables) and student *t*-test (for continuous variables). The associations between *TSER *and *TS 3'-UTR del6 *variant genotypes and breast cancer risk were estimated by computing ORs and their 95% CIs from both univariate and multivariate logistic regression analyses. Linkage disequilibrium (LD) between the two loci of *TYMS *was estimated using the EH algorithm available online [20]. We used the PHASE 2.0 program [21] to infer the haplotype frequencies, based on the observed *TYMS *genotypes. Hardy-Weinberg equilibrium was tested by a goodness-of-fit χ^2 ^test to compare the observed genotype frequencies with the expected frequencies among the control subjects. All the statistical analyses were performed with Statistical Analysis System software (v.9.03e; SAS Institute, Cary, NC).

## Results

The selected characteristics and distributions of *TSER *and *TS 3'-UTR del6 *alleles/haplotypes of the 432 breast cancer cases and 473 cancer-free controls are summarized in Table 1. The frequency-matching on age between cases and controls was adequate as suggested by a non-significant *p *value with the student *t *test. Compared with control subjects, breast cancer patients had a lower age at menarche (*P <*0.0001) and a higher age at having first live birth (*P <*0.0001). Among the 498 postmenopausal subjects, the menopausal age of breast cancer cases was lower than that of the controls (*P *= 0.02). There were 113 (26.2%) breast cancer cases and 95 (20.1%) controls reported a family history of cancer, which was associated with a significantly increased risk for breast cancer (OR = 1.41, 95% CI = 1.03–1.92).

The allele frequencies of *TSER 2R *and *TS 3'-UTR ins6 *were 0.204 and 0.331, respectively, in the controls, while they were 0.202 and 0.310, respectively, in the cases, and the differences were not statistically significant (*P *= 0.987 for *TSER *and *P *= 0.347 for *TS 3'-UTR del6*) (Table 1). In the linkage disequilibrium (LD) analyses, we found that the *TSER *locus was partially in LD with the *TS 3'-UTR del6 *locus (D' = 0.584; R^2 ^= 0.186, *P *<0.001). There were four haplotypes derived from the observed *TYMS *genotypes, but there was no significant difference in the distribution of haplotypes between the cases and controls (*P *= 0.863) (Table 1).

The *TSER *and *TS 3'-UTR del6 *genotype distributions in the cases and controls were shown in Table 2. The genotype frequencies for these two polymorphisms were both in agreement with Hardy-Weinberg equilibrium in the controls (χ^2 ^= 2.26, df = 1, *P *= 0.13 for *TSER *and χ^2 ^= 0.77, df = 1, *P *= 0.38 for *TS 3'-UTR del6*). The *TSER *genotype frequencies were 64.5% (3R/3R), 30.2% (2R/3R) and 5.3% (2R/2R), respectively, among controls, which were not significantly different from those of the cases (64.6% 3R/3R, 30.1% 2R/3R and 5.3% 2R/2R) (*P *= 0.999). Multivariate logistic regression analyses revealed that both 2R/3R and 2R/2R genotypes were not significantly associated with breast cancer risk (adjusted OR = 0.95, 95% CI = 0.70–1.29 for 2R/3R and OR = 1.13, 95% CI = 0.61–2.10 for 2R/2R, respectively), compared with the *TYMS *3R/3R genotype, (Table 2). Likewise, the frequencies of *TS 3'-UTR *del6 genotype were 45.7% (*del6/del6*), 42.5% (*del6/ins6*), and 11.8% (*ins6/ins6*), respectively, in control subjects and 44.9% (*del6/del6*), 48.1% (*del6/ins6*), and 6.9% (*ins6/ins6*), respectively, in breast cancer patients, and the overall difference was statistically significant (*P *= 0.026). Compared with *TS 3'-UTR del6/del6 *wild-type genotype, a significantly reduced risk was associated with *ins6/ins6 *homozygous variant genotype (adjusted OR = 0.58, 95% CI = 0.35–0.97), but it was not related with *del6/ins6 *heterozygous genotype (OR = 1.09, 95% CI = 0.82–1.46). (Table 2)

Because age, menopausal status, age at menarche, age at first live birth and family history of cancer were the well accepted risk factors for breast cancer, we performed stratification analyses by these variables to assess risk modification by the *TYMS *genotypes. We found that reduced breast cancer risk associated with *TS 3'-UTR del6 *variant genotype was more evident in older women (adjusted OR = 0.53, 95% CI = 0.28–1.00), postmenopausal women (adjusted OR = 0.45; 95% CI = 0.22–0.90), individuals with a younger first live birth age (adjusted OR = 0.47; 95% CI = 0.22–1.03) and individuals with a later menarche age (adjusted OR = 0.39; 95% CI = 0.19–0.81) (Table 3). However, we did not find any statistical evidence for any interaction on a multiplicative scale between the variant genotypes and potential risk factors of breast cancer (data not shown).

## Discussion

In this hospital-based case-control study, we investigated the associations of one promoter SNP *TSER *and a *TS 3'-UTR del6 *polymorphism in the *3'-UTR *of the *TYMS *gene with risk of breast cancer in a Chinese population. We found, for the first time, that the *TS 3'-UTR *ins6/ins6 genotype was associated with a significantly decreased risk of breast cancer, and the associations were more evident in older women, postmenopausal women, individuals with a younger first live birth age and individuals with a later menarche age. These findings suggest that the *TS 3'-UTR *variant may contribute to the etiology of breast cancer in our Chinese population.

Although the promoter *TSER *polymorphism was identified in a cis-acting enhancer element of the *TYMS *gene and is thought to affect *TYMS *mRNA expression [16,17,22], several molecular epidemiological studies reported contrary results of the associations between this variant and cancer risk [19,23-34]. In the only one study on breast cancer, the *TSER *variant was found not to contribute to the risk of breast cancer in an Australian population, which is consistent with our current study in Chinese women [27].

The *TS 3'-UTR del6 *polymorphism is located in the *3'UTR *of *TYMS *gene, and the function of this variant has not yet been fully understood. Available limited phenotype studies reported that the wild-type *del6 *allele may be associated with decreased stability of TYMS *in vitro*, lower intratumoral TYMS expression *in vivo *and perhaps with increased risk for cancers [35], which is consistent with our present association study that the variant *ins6 *allele is associated with a decreased breast cancer risk. Because TYMS plays an important role in folate metabolism, which is essential for DNA methylation, synthesis, and repair, our findings provide some evidence for the underline molecular mechanism of the association between folate metabolism and breast cancer susceptibility.

Recently, a few studies have investigated the association between the *TS 3'-UTR del6 *polymorphism and risk of several types of cancer, but the results were mixed [19,23,26,28-30,34]. Graziano *et al *found that the *TS 3'-UTR ins6 *allele was protective against gastric cancer in Caucasians [28]. However, three recent studies reported that the *TS 3'-UTR ins6/ins6 *and/or *del6/ins6 *+*ins6/ins6 *genotypes were associated with a significantly increased risk for gastric cancer, SCCNH and lung cancer [19,23,26], while some other studies did not observe any associations between this polymorphism and cancer risk [29,30,34]. In the present study, we found, for the first time, that the *TS 3'-UTR ins6/ins6 *homozygous variant genotype was associated with a significantly decreased risk of breast cancer in a Chinese population, and this significantly protective effect was more evident in postmenopausal women, individuals with a younger first live birth age or individuals with later menarche age. These findings suggest that *TYMS *polymorphisms may be potential genetic markers of breast cancer risk modified by reproductive hormone levels of the subjects in their lifetime as indicated in menarche age, first live birth age, and menopausal status. However, because of the small sample size in the subgroups, these findings were considered preliminary and need to be validated in further studies with larger sample sizes in these subpopulations. The discrepancies between available association studies in relation to this variant may be due to different etiologies and mechanisms of different cancers, different ethnic backgrounds of populations, uncharacterized environmental exposure factors, and/or potential biases because of the small sample size of related studies.

Genetic polymorphisms often vary between ethnic groups. In the present study in a Chinese population, the frequencies of *TSER *3R/3R, 2R/3R and 2R/2R genotypes were 64.5%, 30.2% and 5.3%, respectively, among 473 control subjects, which were not different from those among the 322 controls in a gastric cancer study in Chinese (63.0% 3R/3R, 33.3% 2R/3R and 3.7% 2R/2R, *P *= 0.447) that we previously reported [19]. However, Marsh *et al *[36] showed a significantly ethnic variation of *TSER *variant that homozygous triple repeat was twice as common in Chinese subjects (67%) as in Caucasians (38%). Similarly, we found that the allele frequency of *TS 3'-UTR *ins6 was 0.331 among the 473 control subjects, which is also consistent with what was reported in Chinese populations (0.320, n = 348 from north China [29], and 0.309, n = 322 from southeast China [19]), but it is much lower than that of Caucasians (65% to 70%)[26,30,34].

The primary shortcoming of this study is the lack of data on detailed dietary intake of folate, plasma or erythrocyte folate levels and its precursors or metabolites such as homocysteine, which limited our inquiry into potential gene–nutrient interactions in breast carcinogenesis. Furthermore, like all other hospital-based case-control studies, inherited biases may lead to spurious findings or false positive results. Because our cases were from hospitals and the controls were from the community, the study subjects may not be fully representative of the general population. However, we applied rigorous epidemiological designs by limiting factors of selecting subjects and performed further statistical adjustment to minimize these potential biases. The agreement with Hardy-Weinberg equilibrium of these two loci of *TYMS *gene and similar allele frequencies of our controls to those reported from other Asian populations suggests that the selection bias would not be substantial in terms of genotype distribution. Finally, although we have 94% statistical power to achieve the main effect of the study, we have very limited power in the stratified analyses because of the small sample size of the subgroups. Therefore, large well-designed studies are warranted to further confirm our findings.

## Conclusion

Our study provides some new evidence that the *TS 3'-UTR ins6/ins6 *genotype may contribute to the etiology of breast cancer. Studies in diverse ethnic populations and simultaneous measurements of detailed data of the *TYMS *genotypes as well as its phenotypes, accompanied by multiple SNPs of other important one-carbon metabolism genes and information of folate intake are warranted to further elucidate gene-gene and gene-environment interactions in susceptibility of breast cancer.

## Abbreviations

TYMS, thymidylate synthase; *TSER*, thymidylate synthase in the 5'-untranslated enhanced region; *TS3'-UTR*, thymidylate synthase in the 3'-untranslated region; OR, odds ratio; CI, confidence interval; SNP, single nucleotide polymorphism; PCR – RFLP, polymerase chain reaction – restriction fragment length polymorphism.

## Competing interests

The author(s) declare that they have no competing interests.

## Authors' contributions

H. S., X. W., F. C. and X. Z. were responsible for the study design, data analyses and interpretation. J. T., J. Q., S. W., X. W. were principally responsible for patients recruitment with assistance from J. G., G. J., J. L. and W. C.. X. Z. and J. G. were responsible for the laboratory work with assistance from G. J. and W. C.. The report was prepared by X. Z. and Z. H., proof read by Q. W. and H. S.. H. S. made the decision to submit the paper for publication and supervised the whole process of the study. All the authors read and approved the final manuscript.

## Pre-publication history

The pre-publication history for this paper can be accessed here:



## Figures and Tables

**Table 1:** Comparison of breast cancer cases and controls by selective characteristics

Variable	Controls^c ^(*n *= 473)	Breast cancer (*n *= 432)	*P *value
Age, yr (mean ± SD)	51.42 ± 11.12	52.09 ± 11.03	0.36
Age at menarche, yr (mean ± SD)	16.40 ± 1.77	15.32 ± 1.92	<0.0001
Age at first live birth, yr (mean ± SD) ^a^	24.49 ± 2.47	25.51 ± 3.45	<0.0001
Age at menopausal, yr (mean ± SD) ^b^	49.79 ± 3.88	48.89 ± 4.26	0.02
BMI	23.69 ± 3.35	23.95 ± 3.55	0.25
Menopausal status			0.74
Premenopausal	210(44.4)	197(45.6)	
Postmenopausal	263(55.6)	235(54.4)	
Family History of Cancer			0.033
Positive	95(20.1)	113(26.2)	
Negative	378(79.9)	319(73.8)	
*TSER*			0.987
3R allele	753(79.6)	688(79.8)	
2R allele	193(20.4)	176(20.2)	
*TS 3'-UTR del6*			0.347
*del6 *allele	633(66.9)	596(69.0)	
*ins6 *allele	313(33.1)	268(31.0)	
Haplotypes of *TSER *and *TS 3'-UTR del6 *polymorphisms			0.863
*3R-del6*	295(62.4)	271(62.6)	
*3R-ins6*	83(17.5)	73(17.0)	
*2R-del6*	25(5.3)	28(6.4)	
*2R-ins6*	70(14.8)	60(14.0)	

^a ^Age at first live birth information was available in 460 controls and 419 breast cancer cases.

^b ^Age at menopausal information was available in 226 controls and 227 breast cancer cases with postmenopausal status.

^c^Three controls carrying the rare alleles of more than three 28 bp repeats (>3R) were excluded in the current analyses.

**Table 2:** *TSER *and *TS 3'-UTR del6 *genotype frequencies among breast cancer cases and controls

Genotype	Controls (N = 473)	Breast cancer (N = 432)	
		
	N (%)	N (%)	^a^Adjusted OR (95% CI)
*TSER*			
*3R/3R *(reference)	305(64.5)	279(64.6)	1.00
*2R/3R*	143(30.2)	130(30.1)	0.95(0.70–1.29)
*2R/2R*	25(5.3)	23(5.3)	1.13(0.61–2.10)
2R/3R and 2R/2R	168(35.5)	153(35.4)	0.98(0.73–1.30)
*TS3'-UTR del6*			
*del6/del6 *(reference)	216(45.7)	194(44.9)	1.00
*del6/ins6*	201(42.5)	208(48.1)	1.09(0.82–1.46)
*ins6/ins6*	56(11.8)	30(6.9)	0.58(0.35–0.97)

^a ^Adjusted for age, age at menarche, menopausal status, BMI and family history of cancer

**Table 3:** Stratified analysis on association between the *TS3'-UTR del6 *polymorphism and risk of breast cancer

Variables	*n *(cases/controls)	TS3'-UTR del6					
		
		n (cases/controls)			^a^Adjusted OR (95% CI)		
			
		*del6/del6*	*del6/ins6*	*ins6/ins6*	*del6/del6*	*del6/ins6*	*ins6/ins6*
Age							
≤ 48	158/195	80/98	69/76	9/21	1.00	1.11(0.70–1.76)	0.65(0.27–1.56)
> 48	274/278	114/118	139/125	21/35	1.00	1.08(0.74–1.58)	0.53(0.28–1.00)
Menopausal status							
Premenopausal	197/210	93/105	90/82	14/23	1.00	1.22(0.80–1.86)	0.75(0.36–1.56)
Postmenopausal	235/263	101/111	118/119	16/33	1.00	1.01(0.68–1.50)	0.45(0.22–0.90)
Age at menarche							
<16	239/152	103/70	117/69	19/13	1.00	1.06(0.68–1.64)	0.87(0.39–1.91)
≥16	193/321	91/146	91/132	11/43	1.00	1.13(0.77–1.65)	0.39(0.19–0.81)
^b^Age at first live birth							
≤ 25	166/226	70/91	85/104	11/31	1.00	1.04(0.67–1.61)	0.47(0.22–1.03)
>25	253/234	116/118	120/95	17/21	1.00	1.24(0.82–1.87)	0.95(0.45–2.03)
Family history of cancer							
No	319/378	143/173	155/165	21/40	1.00	1.07(0.77–1.48)	0.63(0.35–1.13)
Yes	113/95	51/43	53/36	9/16	1.00	1.27(0.69–2.35)	0.45(0.17–1.21)

^a^Adjusted for the other covariates presented in this table in a logistic regression model for each stratum.

^b ^Age at first live birth information was available in 460 controls and 419 breast cancer cases.
